# Outcomes of Genetic Testing-Based Cardiac Rehabilitation Program in Patients with Acute Myocardial Infarction after Percutaneous Coronary Intervention

**DOI:** 10.1155/2022/9742071

**Published:** 2022-08-17

**Authors:** Xing Yu, Yuxuan Fan, Xiaopeng Sun, Xiaojing Wang, Qi Guo, Zhiqing Fan

**Affiliations:** ^1^Department of Rehabilitation Medicine, Shanghai University of Medicine and Health Sciences Affiliated Zhoupu Hospital, Shanghai, China; ^2^Department of Rehabilitation Medicine, Shanghai University of Medicine and Health Sciences, Shanghai, China; ^3^Department of Internal Medicine and Rehabilitation Science, Tohoku University, Sendai-shi, Japan; ^4^Department of Cardiology, Daqing Oilfield General Hospital, Daqing, China

## Abstract

**Objective:**

There can be extreme variability between individual responses to exercise training, and the identification of genetic variants associated with individual variabilities in exercise-related traits could guide individualized exercise programs. We aimed to screen the exercise-related gene sensitivity of patients with acute myocardial infarction after PCI by establishing the gene spectrum of aerobic exercise and cardiopulmonary function sensitivity, test the effect of individualized precision exercise therapy, and provide evidence for the establishment of a precision medicine program for clinical research.

**Methods:**

Aerobic exercise- and cardiopulmonary function-related genes and single-nucleotide polymorphisms (SNPs) were obtained by data mining utilizing a major publicly available biomedical repository, the NCBI PubMed database. Biological samples from all participants underwent DNA testing. We performed SNP detection using Samtools. A total of 122 patients who underwent PCI were enrolled in the study. We screened the first 24 cases with a high mutation frequency for aerobic exercise- and cardiopulmonary function-related genes and the last 24 cases with a low mutation frequency and separated them into two groups for the exercise intervention experiment.

**Results:**

In both the low mutation frequency group and the high mutation frequency group, after 8 weeks of exercise intervention, 6 MWT distance, 6 MWT%, VO_2_/kg at peak, and VO_2_/kg at AT were significantly improved, and the effect in the high mutation frequency group was significantly higher than that in the low mutation frequency group (6 MWT distance: 468 vs. 439, *P*=0.003; 6 MWT%: 85 vs. 77, *P*=0.002, VO_2_/kg at peak: 14.7 vs. 13.3, *P*=0.002; VO_2_/kg at AT: 11.9 vs. 13.3, *P*=0.003).

**Conclusions:**

There is extreme variability between individual responses to exercise training. The identification of genetic variants associated with individual variabilities in exercise-related traits could guide individualized exercise programs. We found that the subjects with a high mutation frequency in aerobic exercise and cardiopulmonary function-related genes achieved more cardiorespiratory fitness benefits in the aerobic exercise rehabilitation program and provided evidence for the establishment of a precision medicine program for clinical research.

## 1. Introduction

Cardiovascular disease is becoming the most common cause of mortality, especially in high-income countries [1]. According to the annual report on cardiovascular health and diseases in China, the prevalence of the cardiovascular disease among Chinese residents has gradually increased, and the prevalence of coronary heart disease among people over 60 years old has reached 27.8% [2]. Since 2005, the mortality of patients with acute myocardial infarction has increased rapidly [2]. Percutaneous coronary intervention (PCI) is an effective treatment to reduce mortality, myocardial infarction, and hospitalization rate of people with the acute coronary syndrome in the treatment of acute myocardial infarction [3, 4].

Although PCI has become the most important revascularization treatment for patients with coronary heart disease, PCI and drug therapy alone cannot continuously and effectively improve the prognosis of patients [5]. It is necessary to prevent the development of coronary heart disease, reduce the recurrence rate and mortality of cardiovascular events, prolong life, and improve the quality of life after discharge [5, 6]. Currently, many international clinical guidelines recommend that patients join an exercise rehabilitation program after PCI [7, 8]. Research shows that exercise rehabilitation can significantly reduce all-cause mortality, cardiovascular disease-related mortality, rehospitalization rate, and the incidence of revascularization, reduce related dysfunction and emotional abnormalities, and increase the quality of life of patients [5, 9]. While exercise is recommended by essentially every major medical organization, it is also recognized that there can be extreme variability between individual responses to exercise training [10].

The idea of personalized medicine has been gaining significant interest since the sequencing of the human genome, and the identification of specific sport- and exercise-related genes is expected to be used for precision sports medicine to provide tailor-made training as well as to select optimal sports and/or other exercise activities for each individual [10–12]. Research has found that sprinters with the RR + RX genotype of the alpha-actinin-3 (ACTN3) gene had significantly faster personal best times for the 100 m race than those with the XX genotype [13]. Thus, the identification of genetic variants associated with individual variabilities in exercise-related traits could guide individualized exercise programs, which is one of the goals of precision medicine [11, 14, 15]. Therefore, we aimed to screen the exercise-related gene sensitivity of patients with acute myocardial infarction after PCI by establishing the gene spectrum of aerobic exercise and cardiopulmonary function sensitivity, test the effect of individualized precision exercise therapy, and provide evidence for the establishment of a precision medicine program for clinical research.

## 2. Methods

### 2.1. Data Mining of the Gene Set

We utilized a major publicly available biomedical repository, the NCBI PubMed database, for data mining. Search strategies were combined as follows: (“athletic performance” OR “physical performance” OR “elite athlete” OR “athletic status” OR “endurance performance” OR “aerobic exercise” OR “strength training”) AND (genes OR gene OR loci OR locus). Database searching retrieved a total of 951 studies. Information on a total of 111 exercise-related SNPs and 76 exercise-related genes was obtained after analysis by a text mining program (see Table S1). Our text mining program consisted of five steps: (1) Document searching and formatting, in which keywords were used to search documents and organize documents into XML format. (2) Gene mentions tagging using ABNER software to describe and locate genes [16]. (3) Conjunction resolution, in which the description of extracted genes, such as the “STAT3/5 gene,” was resolved into the STAT3 gene and STAT5 gene. (4) Gene name normalization based on the Entrez database; because the names of genes in the free text were confusing, it was necessary to unify the gene descriptions in the article into official gene symbols to facilitate analysis and comparison. The gene symbol was based on the Entrez gene database of the NCBI. (5) Statistical analysis, in which the frequency of each gene was determined. The higher the frequency of the gene, the greater the possibility that the gene was related to the disease. The total number of documents in the PubMed database was defined as N. The frequency of independent occurrence of genes and corresponding diseases in the PubMed literature database was recorded as *m* and *n*, respectively. Supposing that the number of simultaneous occurrences of gene disease is *k*, we can calculate the probability of more than *k* power co-citations under completely random conditions through hypergeometric distribution, as follows:

(1) $$\begin{array}{c} p=1-\sum_{i=0}^{k-1}p\left(i|n,m,N\right), \end{array}$$

and

(2) $$\begin{array}{c} p\left(i|n,m,N\right)=\frac{n!\left(N-n\right)!m!\left(N-m\right)!}{\left(n-i\right)!i!\left(n-m\right)!\left(N-n-m+i\right)!N!}. \end{array}$$

**Figure 1:**
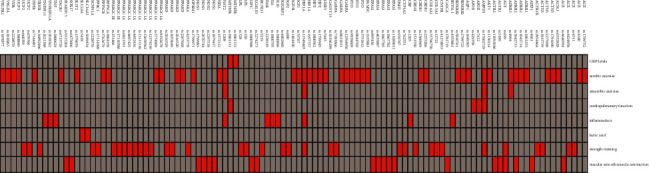
Heatmap of 111 SNP.

Through the classification of aerobic exercise- and cardiopulmonary function-related genes involved in this exercise program, 36 aerobic exercise- and cardiopulmonary function-related genes and 45 related SNPs were obtained (see Figure 1).

The red color indicates that the SNP appears, and grey indicates that it does not appear.

### 2.2. Subjects and Groups

Patients were recruited from DAQING Oilfield General Hospital. In the inclusion criteria, patients were recruited from Daqing Oilfield General Hospital. The inclusion criteria were as follows: (1) all patients were treated with PCI for the first time and whose Killip class was I-II; (2) all patients were treated within 6 hours after the onset of disease, and (3) clinical data and imaging data during the treatment period were complete, and there were no missing data. The exclusion criteria were as follows: (1) exercise-induced syncope or ventricular arrhythmias; (2) inability to exercise and walk owing to comorbidities; (3) suffering from end-stage diseases such as malignancies; (4) severe complications such as pulmonary edema, severe arrhythmia, or cardiogenic shock; and (5) suffering from mental illness or family history of mental illness. All percutaneous coronary interventions were performed by the same team [17]. A total of 122 patients who underwent PCI after acute myocardial infarction were enrolled in the study. Informed written consent was obtained from all participants. Biological samples from all participants underwent DNA testing. The DNA of the 122 subjects was extracted using a Universal Cylindrical Genome Extraction Kit (KangWei Century, CW2298 M). Agarose gel electrophoresis was used to analyze the degree of DNA degradation and the presence of impurity bands and RNA and protein contamination in the 122 subjects. The quality of the DNA was determined by a NanoDrop ND-2000 ultramicro spectrophotometer. Biological samples were gene sequenced using the Illumina HiSeq PE150. Quality control (QC) analyses were performed on sequenced Reads. Mapping reads to a reference after QC. The sequencing results were compared with the reference genome using BWA software which mainly uses the location comparison between a large number of short fragments after second-generation sequencing and the reference genome. We performed SNP genotyping through SAMtools software [18, 19]. The regional distribution statistics of 45 aerobic exercise- and cardiopulmonary function-related SNP loci of the 122 samples are shown in Table 1.

**Figure 2:**
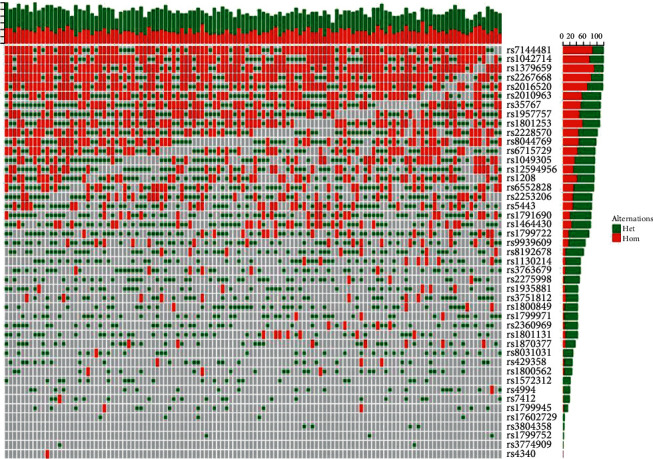
Panorama of 45 SNP gene mutations in subjects.

We drew a panorama of 45 SNP genotypes from 122 samples, screened the first 24 cases with high mutation frequency and the last 24 cases with low mutation frequency, and divided them into two groups for the exercise intervention experiment, as shown in Figure 2. There were no statistically significant differences between the 2 groups of patients regarding general data such as age, sex, body mass index (BMI), Killip classification, coronary lesions, and comorbidities such as hypertension or diabetes (*P* > 0.05); the groups were comparable.

### 2.3. Procedures

**Figure 3:**
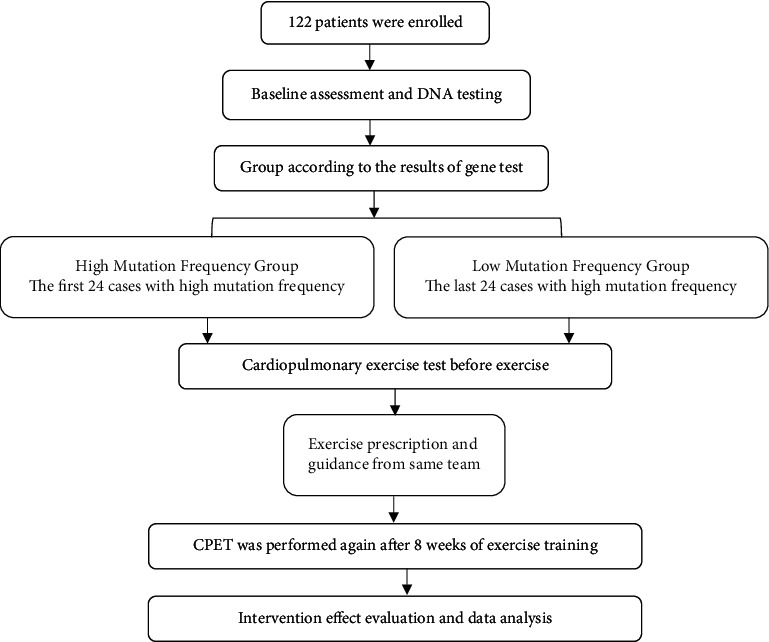
Intervention flow chart.

A total of 122 patients who underwent PCI after acute myocardial infarction were enrolled in the study, and the first 24 cases with a high mutation frequency in aerobic exercise and cardiopulmonary function-related genes and the last 24 cases with a low mutation frequency were screened into two groups for the exercise intervention experiment. The flow chart is shown in Figure 3. After PCI treatment, all patients underwent blood pressure regulation, sedation, and other treatments, and symptomatic care, such as oxygen and medication administration, was also delivered. These two groups were given an exercise prescription and guidance from the same team. Each exercise period consisted of warm-up exercises for 5 min (20–40% of VO_2_ max), followed by moderate-intensity continuous aerobic exercise (50–60% of VO_2_ max), light exercise for 40 min, and a 5-min cooldown; the regimen had a Borg rating of 11–13. The exercise was performed for 50 minutes each time, 4 times/week, for a total of 8 weeks. The exercise was advised to be halted if any of the following occurred: (1) chest pain, dyspnoea, or dizziness during or after exercise; (2) heart rate fluctuation >30 beats/min; (3) blood pressure >200/100 mmHg or systolic blood pressure increase >30 mmHg or decrease >10 mmHg; (4) electrocardiogram monitoring during exercise showed ST-segment depression  ≥0.1 mV or elevation  ≥0.2 mV; or (5) severe arrhythmia during or after exercise. This study was approved by the DAQING Oilfield General Hospital ethics committee.

### 2.4. Outcome Measures

Cardiopulmonary exercise testing with respiratory gas analysis was performed using the individualized ramp protocol recommended by the American Heart Association [20]. The specific protocol was as follows: 0 W: rest for 1 min; 0 W: warm-up for 2 min; treadmill intensity started at 5 W. Thereafter, according to the exercise ability of the subjects, the intensity was increased by 15–25 W per minute until the subjects reached the outcome measures at 8–12 minutes. Calibration was performed before each testing period. Peak O_2_ utilization (VO_2_ peak) was defined as the highest VO_2_ value (without reaching an oxygen uptake steady-state plateau), achieved at individual maximum load during incremental exercise testing [20]. During CPET, data on the subjects' static electrocardiogram and static lung function (vital capacity/maximum ventilation) were individually collected. The indications for termination of CPET in our study were following the scientific statement from the American Heart Association [21]. The 6-minute walk test (6 MWT) was used in this study. The procedures and indications for termination of the 6 MWT were performed according to the recommendations of the American Thoracic Society [22]. The participants walked for 6 minutes along an indoor 30-m corridor, and the distance walked was recorded for analysis.

### 2.5. Statistical Analyses

Continuous variables are expressed as the mean ± SD or median and were compared using a two-sided independent samples *t* test. Frequency data were compared between the groups using the chi-square test. A value of *p* < 0.05 was considered statistically significant. Statistical analysis was performed using *R* version 4.0.5.

## 3. Results

The average age of the high mutation frequency group was 52.76 ± 7.74 years, and that of the low mutation frequency group was 50.76 ± 9.34 years. There was no significant difference in sex, age, or exercise ability indices, including the 6 MWT, VO_2_/kg at peak, and VO_2_/kg at AT, between the low mutation frequency group and the high mutation frequency group, as shown in Table 2.

After 8 weeks of exercise training, the 6-minute walk test distance (468 vs. 439, *P*=0.003), 6 MWT% (85 vs. 77, *P*=0.002), VO_2_/kg at peak (14.7 vs. 13.3, *P*=0.002), and VO_2_/kg at AT (11.9 vs. 13.3, *P*=0.003) in the high mutation frequency group were significantly higher than those in the low mutation frequency group, as shown in Table 3.

Through the independent samples *t*-test analysis of the results in the group, it was found that the 6-minute walk test distance (388 vs. 468, *P* < 0.01), 6 MWT% (68 vs. 85, *P* < 0.01), VO_2_/kg at peak (12.1 vs. 14.7, *P* < 0.01) and VO_2_/kg at AT (9.1 vs. 11.9, *P* < 0.01) in the high mutation frequency group after 8 weeks of training were significantly higher than those before training, as shown in Table 4.

Through the independent samples *t*-test analysis of the results in the group, it was found that the 6-minute walk test distance (385 vs. 439, *P* < 0.01), 6MWT% (67 vs. 77, *P* < 0.01), VO_2_/kg at peak (12.4 vs. 13.3, *P* < 0.01), and VO_2_/kg at AT (9.3 vs. 10.1, *P* < 0.01) in the low mutation frequency group after 8 weeks of training were significantly higher than those before training, as shown in Table 5.

**Figure 4:**
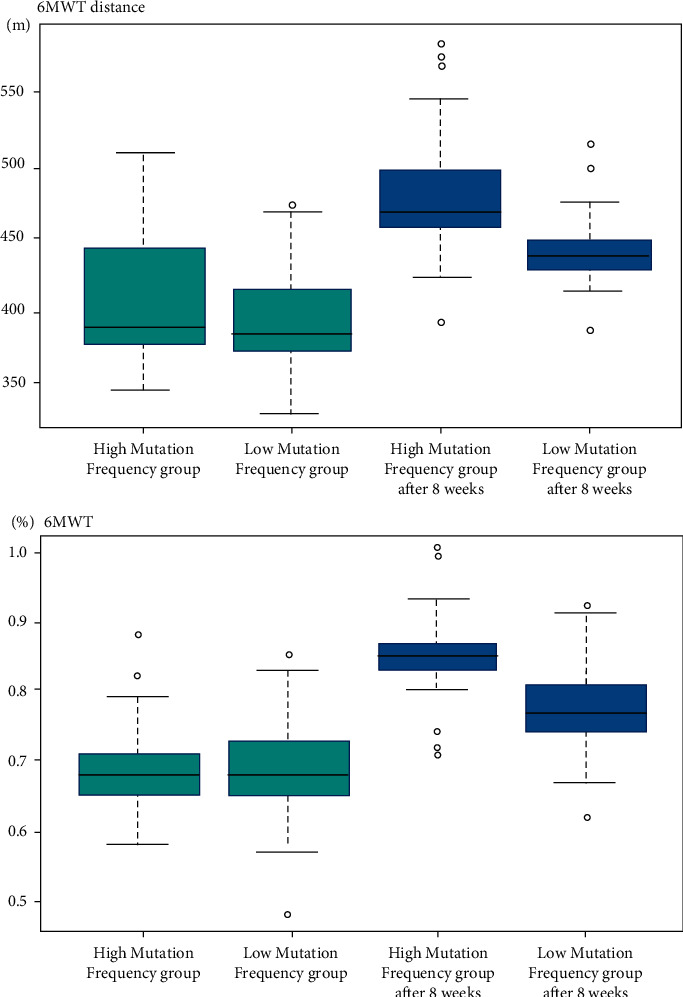
6MWT distance and 6MWT% before and after the 8-week exercise intervention.

In both the low mutation frequency group and the high mutation frequency group, after 8 weeks of exercise intervention, 6MWT distance (low mutation frequency group: 385 vs. 439, *P* < 0.01; high mutation frequency group: 388 vs. 468, *P* < 0.01) and 6MWT% (low mutation frequency group: 67 vs. 77, *P* < 0.01; high mutation frequency group: 68 vs. 85, *P* < 0.01) were significantly improved, and the effect in the high mutation frequency group was significantly higher than that in the low mutation frequency group (6 MWT distance: 468 vs. 439, *P*=0.003; 6 MWT%: 85 vs. 77, *P*=0.002), as shown in Figure 4.

**Figure 5:**
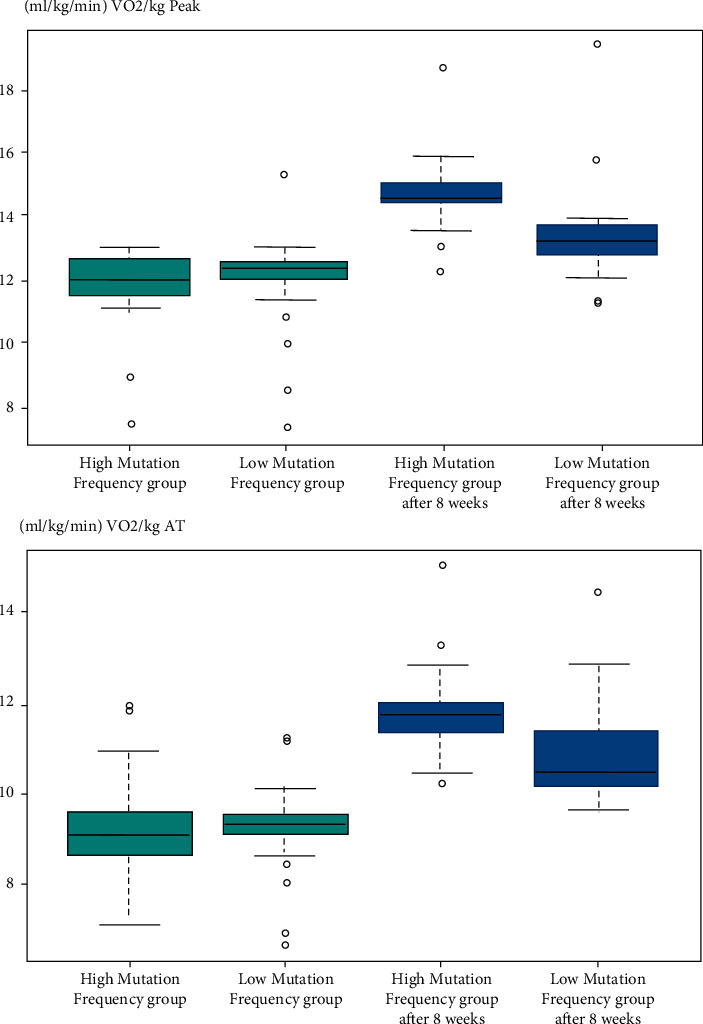
VO_2_/kg at peak and VO_2_/kg at AT before and after the 8-week exercise intervention.

After 8 weeks of exercise training, VO_2_/kg at peak (low mutation frequency group: 12.4 vs. 13.3, *P* < 0.01; high mutation frequency group: 12.1 vs. 14.7, *P* < 0.01) and VO_2_/kg at AT (low mutation frequency group: 9.3 vs. 10.1, *P* < 0.01; high mutation frequency group: 9.1 vs. 11.9, *P* < 0.01) were significantly improved in both the low mutation frequency group and the high mutation frequency group, and the effect in the high mutation frequency group was significantly higher than that in the low mutation frequency group (VO_2_/kg at peak: 14.7 vs. 13.3, *P*=0.002; VO_2_/kg at AT: 11.9 vs. 13.3, *P*=0.003), as shown in Figure 5.

## 4. Discussion

As previous studies have suggested, genetic modifiers have been identified from the study of affected patient populations to identify common genomic variations. In precision medicine, these findings could provide the most useful results in terms of applicability in the clinic [10]. Findings from numerous investigations demonstrate extraordinary interindividual variability in response to a standard dose of exercise [23], and the issue of individual response to treatment is one of the most important in exercise medicine. In our study, we tried to select patients with more mutations in aerobic exercise and cardiopulmonary function sensitivity genes through gene mutation detection to carry out an aerobic exercise intervention to detect whether they would obtain more benefits compared with a low mutation frequency group, which is consistent with some previous studies [11, 14]. Previous twin and familial studies suggest that there is moderate heritability of “sport and exercise-related traits” [22], thus, the identification of genetic variants determining variabilities in sport and exercise-related traits may offer significant benefits to athletes and the general population [10,11]. We found that the group with a high mutation frequency for aerobic exercise- and cardiopulmonary function-related genes gleaned more benefits from the 8-week aerobic exercise rehabilitation program (MWT distance: 468 vs. 439, *P*=0.003; 6MWT%: 85 vs. 77, *P*=0.002, VO_2_/kg at peak: 14.7 vs. 13.3, *P*=0.002; VO_2_/kg at AT: 11.9 vs. 13.3, *P*=0.003).

Research has found considerable interindividual responses to a single-dose exercise program for maximal oxygen uptake (VO_2_ max), which is achieved at the individual maximum load during incremental exercise testing [23]. The concept of genetic variation being associated with trainability has been extensively studied in relation to peak VO_2_, potentially explaining up to 50% of the variability in the change in peak VO_2_ after endurance training [24, 25]. Many previous large-scale trials and meta-analyses used the 6 MWT and peak VO_2_ to demonstrate the physical and physiological benefits of routine cardiac rehabilitation [5, 26, 27]. VO_2_ max describes the maximum ability of a whole organism to transport oxygen from the air to the tissues and especially the exercising skeletal muscles [24, 28]. The maximal amount of O_2_ per unit of time that can be delivered to peripheral organs, including skeletal muscle, where it is used to sustain muscular contraction at peak exercise, is considered the gold standard measure of cardiorespiratory fitness [29, 30]. Peak or maximum cardiac output and total body hemoglobin mass seem to predominate as determinants of max VO_2_. Cardiorespiratory fitness is closely associated with all-cause mortality and cardiovascular mortality. Thus, we will further carry out exercise intervention projects for cardiovascular disease patients to reduce the incidence and mortality of heart failure.

In addition, human athletic performance has long been assumed to be polygenic. In addition to single-nucleotide variants in the gene regions, other types of genomic variation, such as structural variation and variants in noncoding RNA, may also contribute to the complexity of the athletic phenotype [31, 32]. Given that exercise is polygenic within a given organ and affects multiple organ systems, there are likely other undetermined adaptations that do respond to exercise [33]. Studies have suggested that subjects show improvements in oxidative enzyme activities in muscles even in the group that did not show an increase in VO_2_ max in response to aerobic exercise [34]. The evaluation indicators included in our study are limited; thus, failure to improve one specific phenotype is not reason enough to cease or fail to recommend or prescribe exercise because VO_2_ max does not increase. We will screen to carry out exercise therapy and interventions efficiently and accurately for cardiovascular disease patients. In addition to clinical efficacy and safety, the costs and cost-effectiveness of cardiac rehabilitation need to be considered with the growing cost pressures on healthcare systems across the world [35]. Previous studies concluded that cardiac rehabilitation was cost-effective compared with no cardiac rehabilitation (incremental cost-effectiveness ratios (ICERs) ranged from US$1,065 to US$71,755 per quality-adjusted life-year (QALY)), and exercise intervention in cardiac rehabilitation appears to cost-effective, though uncertainty was high [36]. Thus, optimal tailored medical therapies for the individual based on the individual's complete clinical and risk profiles which include their genomic information may revolutionize healthcare by substantially enhancing the efficacy of treatment with a promise to significantly reduce the costs associated with healthcare provision [37].

On the other hand, although a large number of studies have been conducted to identify sport- and exercise-related genes, the findings are mostly inconclusive because of a lack of replication, which is caused by the small sample sizes [11, 38]. Similarly, the sample size in our study is limited. Common SNPs associated with polygenic traits (including sport- and exercise-related traits) generally show a modest OR of 1.1–1.5 [39], and each physiological marker of performance is a complex trait regulated by a network of genes and pathways [11, 40]. A study suggests that a sample size of less than 1000 is still insufficient despite a well-standardized intervention protocol and precise phenotyping [11], therefore, both a large sample size and precise phenotyping are necessary to reduce the SE and increase statistical power to detect a significant SNP-trait association [11]. In addition, although the variability in individual training responses to improved maximal aerobic capacity after exercise-based cardiovascular rehabilitation exists in both healthy subjects and patients with established cardiovascular disease [41], but the interaction between gene variants and disease-modifying factors adds to the complexity, it is unclear whether genomic predictors of training response are the same in healthy and at-risk or diseased populations, and this study lacks the comparison of cardiac rehabilitation effect between normal people and patients with acute myocardial infarction after PCI. In the future, we will plan further exercise intervention projects combined with large-scale gene testing and screening to carry out exercise therapy and interventions efficiently and accurately for cardiovascular disease patients, reduce the incidence and mortality of heart failure, and provide evidence for clinical research.

## 5. Conclusions

Cardiovascular disease is a major public health problem worldwide. PCI is an effective treatment to reduce mortality, myocardial infarction, and hospitalization rate of the acute coronary syndrome in the treatment of acute myocardial infarction. While exercise is recommended by essentially every major medical organization, it is also recognized that there can be extreme variability between individual responses to exercise training. We found that the intervention group with a high mutation frequency in aerobic exercise- and cardiopulmonary function-related genes achieved more benefits in the 8-week aerobic exercise rehabilitation program. Thus, we will plan further exercise intervention projects combined with large-scale gene testing and screening to carry out exercise therapy and interventions efficiently and accurately for cardiovascular disease patients, reduce the incidence and mortality of heart failure, and provide evidence for clinical research.

## Figures and Tables

**Table 1:** Regional distribution of 45 SNP loci.

Func.refGene^1^	Number
Exonic^2^	16
ncRNA_intronic^3^	2
Intronic^4^	15
UTR3^5^	3
UTR5^6^	5
Downstream^7^	1
Intergenic^8^	3
Total^9^	45

^1^The functional region where the mutation site is located; ^2^exonic region; ^3^noncoding RNA intron region; ^4^inner subregion; ^5^3′ UTR area; ^6^5′ UTR area;^7^1 KB region downstream of transcription termination site; ^8^gene spacer region;^9^ Total SNP.

**Table 2:** Comparison of baseline indexes between the high mutation frequency group and the low mutation frequency group.

	High mutation frequency group (*n* = 24)	Low mutation frequency group (*n* = 24)	*P* value
Age (Y)	52.76 ± 7.74	50.76 ± 9.34	0.41
Gender			0.63
Male %	88	92	
Female %	12	8	
6 MWT (m)	388	385	0.16
6 MWT%	68	67	0.77
VO_2_/kg PEAK (ml/kg/min)	12.1	12.4	0.68
VO_2_/kg AT (ml/kg/min)	9.1	9.3	0.91

6 MWT: 6-minute walk test. AT: anaerobic threshold.

**Table 3:** Comparison of exercise ability indices after 8 weeks of exercise intervention.

	High mutation frequency group (*n* = 24)	Low mutation frequency group (*n* = 24)	*P* value
6 MWT (m)	468	439	0.003
6 MWT%	85	77	0.002
VO_2_/kg PEAK (ml/kg/min)	14.7	13.3	0.002
VO_2_/kg AT (ml/kg/min)	11.9	10.1	0.003

**Table 4:** Comparison of indices in the high mutation frequency group before and after the 8-week exercise intervention.

	High mutation Frequency group before (*n* = 24)	High mutation Frequency group after (*n* = 24)	*P* value
6MWT (m)	388	468	<0.01
6MWT%	68	85	<0.01
VO_2_/kg PEAK (ml/kg/min)	12.1	14.7	<0.01
VO_2_/kg AT (ml/kg/min)	9.1	11.9	<0.01

**Table 5:** Comparison of indices in the low mutation frequency group before and after the 8-week exercise intervention.

	Low mutation frequency group before (*n* = 24)	Low mutation frequency group after (*n* = 24)	*P* value
6 MWT (m)	385	439	<0.01
6 MWT%	67	77	<0.01
VO_2_/kg PEAK (ml/kg/min)	12.4	13.3	<0.01
VO_2_/kg AT (ml/kg/min)	9.3	10.1	<0.01

## Data Availability

The data used to support the findings of this study are available from the corresponding author upon request.

## Supplementary Materials

Supplementary Materials Table S1. A total of 111 exercise-related single nucleotide polymorphisms (SNP) and 76 exercise-related genes were obtained after analysis by a text mining program.
